# FIBFLO – a study design for comparing the effects of diets on the microbiome and its metabolism: β-glucan or not?

**DOI:** 10.1080/16512235.2017.1281946

**Published:** 2017-02-13

**Authors:** E. Norin, L. Engstrand, P. Hellström, L. Martin Marais, T. Midtvedt, R. Möllby, I. Ernberg

**Affiliations:** ^a^ The FIBFLO-Group, Department of Microbiology, Tumor and Cell Biology, Karolinska Institutet, Stockholm, Sweden; ^b^ Department of gastroenterology & hepatology, Akademiska Hospital, Uppsala, Sweden; ^c^ Lantmännen, Stockholm, Sweden

It has been established that the human gut microbiome is commensal, helps to digest our food, and is involved in metabolic processes in the gut and in the development and maintenance of our immune systems.[1] The microbiome and its genes process the primarily indigested food we eat and deliver metabolites, which frequently are taken up in our blood and by our microbiota. An estimated 10% of the small metabolites in our blood plasma are derived from microbial metabolism. However, only recently there has been a general acceptance of its importance for human development in health and disease. This depends largely on the availability of high-throughput sequencing methods which allows efficient and cost-effective tools to identify parts of the members of the gut microbiome and how they might be affected by environmental perturbations.[2–4] The human microbiome is a promising asset as an early biomarker for disease risks and a target for dietary and non-invasive therapeutic interventions.

Among all factors which, under physiological conditions, might affect the composition and function of gut microbiota (GM), diet is by far the most important and is also the easiest factor to use in order to manipulate the GM.[5–7]

In this context, oats represent a unique and challenging dietary ingredient. Much attentions have been focused on its content of β-glucan which is a linear mixed glucose polymer with glucose residues linked via beta-1–3 (about 30%) and beta-1–4 linkages (around 70%).[8] Results from *in vitro* fermentation studies [8,9] as well as *in vivo* animal studies demonstrate effects of oats on GM.[8–12] In spite of all the promising health-promoting effect of oats consumption, it is reasonable to assume that multifaceted studies in humans may uncover new human-health benefits of oats consumption, maybe also on an individual level.

The present investigation was designed as a pilot study for establishing a multi-disciplinary approach, including collection and evaluation of anthropomorphic, microbiomic, metabolomics, immunological and gut-related functional data in a cohort of healthy adult volunteers daily receiving a pre-made meal with or without oat β-glucan for a defined period of time in a double-blinded cross-over study.[13,14]

The aim was to evaluate a test system of intestinal microbiological, biochemical and immunological parameters to determine the effects of β-glucan fiber on composition and function of human intestinal microbiota in healthy volunteers.

Twenty males were recruited by advertisement. The reason for including only men in the study was to try to avoid hormone influences and other external factors in this first screening. The study was a double blinded investigation including 10 volunteers per group. Initially, they were examined by a clinician (PH) for health status including absence of factors that could influence microbiota functions, e.g. antimicrobials. Before, during and after the dietary intervention, blood, urine and feces were collected for analyses and at the same time, the participants swallowed a ‘smart pill’.[15] Moreover, the participants filled in questionnaires regarding hunger and satiety, Bristol scale, gastrointestinal symptoms and wellbeing. Each study period lasted for two weeks.

**Table:** 

Diet A	Diet B
Diet B	Diet A

The food producer provided the participants with preprepared bags with products with high or low levels of β-glucan content and advised how to prepare the food during the study. Blood, serum and urine samples are stored pending analyses.

The fecal samples will be used for culturing microbes [13] for analysis of several biochemical markers, such as short chain fatty acid patterns, conversion of cholesterol and bilirubin, degradation of mucin and levels of tryptic activity,[14] and several immunological factors. Metabolomics analyses will be performed as well as metagenomics analyses.
